# Ge‐Based Visible‐Infrared Bipolar Floating‐Gate Phototransistor for Broad‐Spectrum Retinal Bionics

**DOI:** 10.1002/advs.202512649

**Published:** 2025-09-19

**Authors:** Qiancui Zhang, Yichi Zhang, Xie Liu, Ningning Zhang, Tian Miao, Huiyong Hu, Liming Wang, Zhangming Zhu

**Affiliations:** ^1^ Key Laboratory of Analog Integrated Circuits and Systems (Ministry of Education) School of Integrated Circuits Xidian University Xi'an 710071 China; ^2^ Laboratory of Analog Integrated Circuits Hangzhou Institute of Technology Xidian University Hangzhou 311231 China

**Keywords:** bipolar photoresponse, broad‐spectrum, floating‐gate, human retina, WSe_2_ photodetector

## Abstract

Bipolar cells play a crucial role in perceiving, transmitting, and processing visual information by effectively segregating visual signals into ON and OFF pathways. However, conventional visual signals are predominantly confined to the visible (Vis) wavelength range, which significantly restricts the potential applications of bipolar cells. Here, a germanium (Ge)‐based floating‐gate tungsten diselenide (WSe_2_) phototransistor capable of mimicking broad‐spectrum bipolar cell functionality is proposed, which extend the utility from artificial retinal systems to advanced image processing applications. The floating gate of the phototransistor can non‐volatilely storage electrons/holes, modulating the WSe_2_ channel to exhibit bipolar characteristics. Under distinct bottom gate voltage pulse modulations, the bipolar WSe_2_ channel interfaces with multilayer graphene (MLG) to form Schottky built‐in electric fields with opposing directions, enabling bipolar photoresponse under visible light illumination. Meanwhile, by leveraging the near‐infrared (NIR) light absorption of Ge and the photogating effect, the device demonstrates NIR bipolar photoresponse. Based on the unique dual‐band bipolar photoresponse characteristics of this single phototransistor, the neural biological functions of bipolar cells in the human retina are successfully emulated, and an image sharpening processing based on convolutional operation is demonstrated. This advancement significantly enhances the potential of visual bionic chips for applications in vehicles or robots.

## Graphical abstract

A germanium (Ge)‐based floating‐gate tungsten diselenide (WSe_2_) phototransistor capable of mimicking broad‐spectrum bipolar cell functionality is proposed. Under different bottom gate voltage pulse modulations, the device exhibits bipolar response under both visible and near‐infrared light illumination. Based on the unique dual‐band bipolar photoresponse characteristics of this single phototransistor, the neural biological functions of bipolar cells in the human retina is emulated.

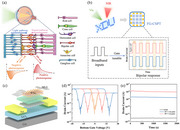

## 1 Introduction

Vision, essential for humans to perceive their environment and gather information, is crucial in areas like environmental monitoring, smart homes, and autonomous driving.^[^ ^1^ ^]^ In traditional image sensing systems, optical signals are captured by pixel arrays, converted to electrical signals, and then digitized for storage and processing. Despite their maturity, these systems often face delays and high power consumption due to their complexity, resulting in inefficient data handling.^[^ ^2^ ^]^ To address these challenges, artificial bionic retinal systems offer a promising solution for high‐performance image perception and processing. In such systems, multifunctional layers and neural networks, organized by nerve cells, not only receive and transmit visual signals but also perform preliminary signal preprocessing. This eliminates redundant data, reduces delays caused by transmitting large volumes of information, and accelerates in‐system computation.^[^ ^3^ ^]^

Bipolar cells, as intermediate neurons in the retina, perform a pivotal function in connecting the upstream and downstream neural circuits. They receive input signals from photoreceptors, integrate them, and transmit the signals to amacrine cells and ganglion cells, thereby shunting visual signals into ON and OFF pathways.^[^ ^4^ ^]^ Over the past few decades, various optoelectronic sensors have been developed for artificial bionic retinal systems.^[^ ^1^, ^5^ ^]^ Among these, 2D materials have gained significant attention due to their exceptional electronic and optical sensing and processing capabilities.^[^ ^6^ ^]^ Notably, tungsten diselenide (WSe_2_) has emerged as a promising candidate, owing to its superior optoelectronic properties and inherent bipolar characteristics.^[^ ^7^ ^]^ Recent studies have developed WSe_2_‐based photodetectors and multi‐gate‐controlled composite structures, highlighting their potential for artificial bionic retinal systems.^[^ ^3^, ^8^ ^]^ However, few devices mimic the bipolar photoresponse of neurons, and these responses are often limited to specific optical signals at different wavelengths.^[^ ^9^ ^]^ While gate‐controlled structures are favored for their multifunctionality, most rely on constant gate voltages, resulting in high power consumption.^[^ ^2^, ^10^ ^]^ Thus, a low‐power photodetector with bipolar photoresponse to identical optical signals remains a challenge.

In this work, we propose a Ge‐based WSe_2_ floating‐gate composite structure phototransistor (FG‐CSPT) capable of sensing and processing optical signals. By applying pulsed bottom gate voltage of different polarities, electrons/holes tunnel through the dielectric layer and are stored in the floating gate, modulating the carriers distribution in the channel and thus its conductive properties.^[^ ^8^, ^11^ ^]^ Therefore, oppositely oriented Schottky barriers are formed at the MLG contact. This mechanism influences photogenerated carriers transport, achieving bipolar photoresponse to visible light signals. Notably, the incorporation of the 3D semiconductor material Ge extends the device's optical response from visible light to near‐infrared (NIR) light. Leveraging the NIR absorption of the Ge‐based substrate and the photogating effect, the device exhibits bipolar photoresponse in the infrared range. This capability enables low‐power bipolar photoresponse across a broad Vis‐NIR spectrum, effectively mimicking and exceeding the biological functions of neurons in the mammalian retina. This breakthrough offers a novel approach for developing high‐performance, multifunctional vision bionic chips for applications in vehicles or robots.

## 2 Results and Discussion

**Figure 1:**
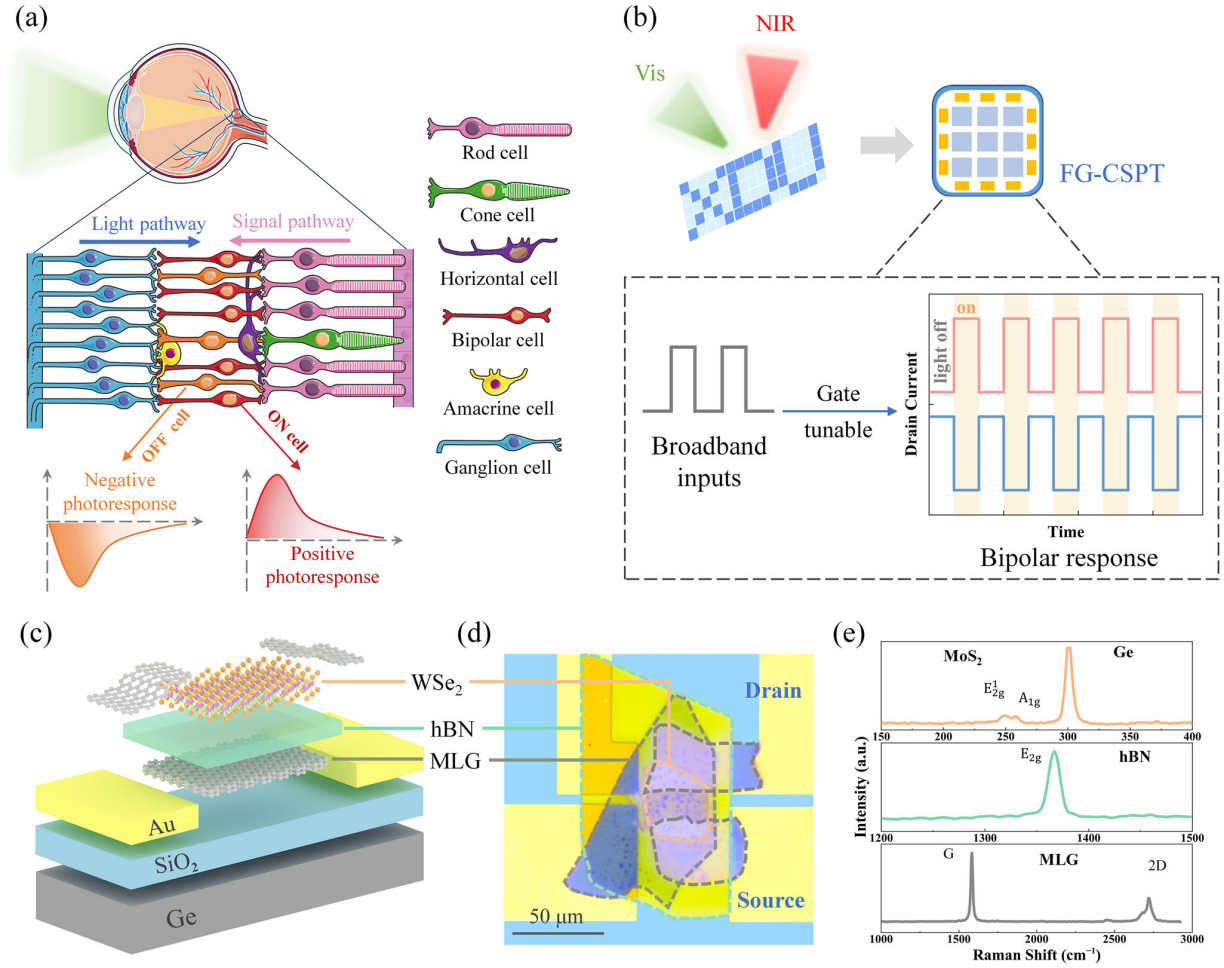
Design and characteristics of the floating‐gate composite structure phototransistor. a) Simplified schematic of signal transmission process in the human retinal system and working mechanism of ON and OFF bipolar cells in photoresponse. b) Conceptual schematic of FG‐CSPT with image sensing and processing processes. The device not only receives external image information, but also pre‐processes and modulates signals by programming its photoresponse through gate voltage. c) Schematic of the device structure. d) Optical microscope image of the phototransistor (orange, green and grey outlines for WSe_2_, hBN, and MLG, respectively). e) Raman spectrum of the device.

A typical mammalian retina consists of various distinct types of neuronal cells, including photoreceptors (rod cells and cone cells), horizontal cells, bipolar cells, amacrine cells, and ganglion cells, as shown in **Figure** **1**a. Photoreceptors convert optical signals into electrical signals, while bipolar cells receive signals from the photoreceptors and transmit them to downstream ganglion cells, thus facilitating rapid perception in the brain.^[^ ^12^ ^]^ Bipolar cells are further categorized into ON‐cells and OFF‐cells, which exhibit positive or negative response to light stimuli, respectively.^[^ ^13^ ^]^ Inspired by the mammalian retinal system, we propose a bionic floating‐gate composite structure phototransistor. Figure 1b illustrates the core design concept, in which optical signal sensing and processing are integrated within a unified framework. The FG‐CSPT can detect optical signals and exhibit bipolar response characteristics under bottom gate voltage pulse modulation. To emulate the biological properties of photoreceptors and bipolar cells in the retina, we fabricated a symmetrical van der Waals (vdWs) stacked WSe_2_ floating‐gate phototransistor. Figure 1c shows a schematic diagram of FG‐CSPT prepared on a SiO_2_ (300 nm)/p‐Ge substrate, where the floating gate MLG, hexagonal boron nitride (hBN), and WSe_2_ are used as storage layer, tunneling layer, and conducting channel, respectively. Due to its high carrier mobility, MLG flakes are adopted as drain and source terminals of the phototransistor, enabling high‐performance optoelectronic devices. Under the bottom gate voltage pulse modulation, carriers in the channel tunnel through hBN to the floating gate MLG, electrostatic doping the WSe_2_ layer. A detailed fabrication process of the device is described in the Experimental Methods section. The optical microscope image of FG‐CSPT is shown in Figure 1d, and the outlines of the materials are marked with colors. Figure 1e characterizes the Raman spectrum of WSe_2_ FG‐CSPT. All typical Raman properties of WSe_2_ ($E_{2g}^{1}$ peak at 250.1 cm^−1^, A_1g_ peak at 257.1 cm^−1^), Ge (Ge‐Ge peak at 301.8 cm^−1^), hBN (E_2g_ peak at 1367.1 cm^−1^) and MLG (G peak at 1581.6 cm^−1^, 2D peak at 2721.8 cm^−1^) can be observed from the results.^[^ ^14^ ^]^ The thicknesses of materials are determined respectively using an atomic force microscope (AFM) (Figure S1, Supporting Information).

**Figure 2:**
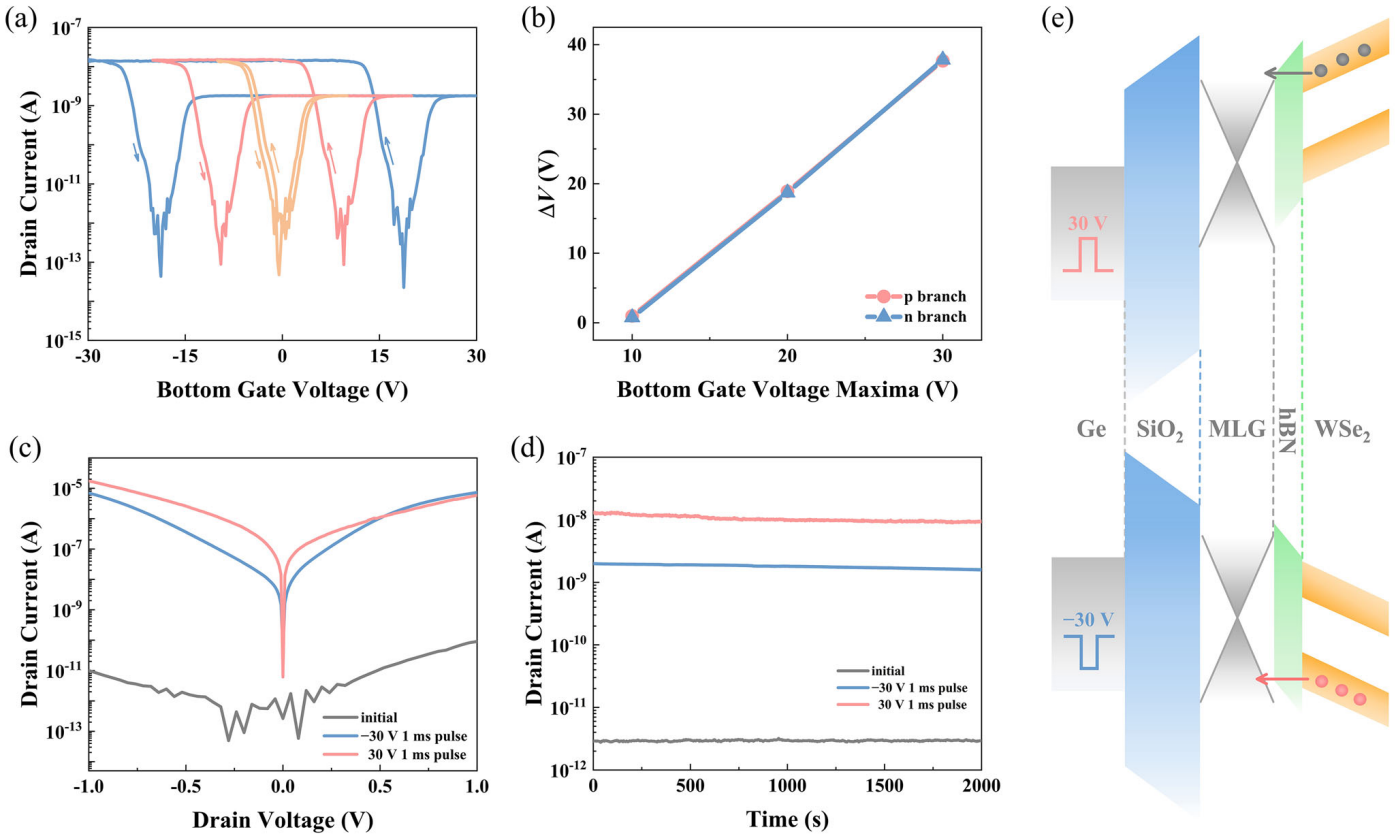
Electrical WSe_2_ FG‐CSPT performance. a) Transfer curves of the device at bottom gate voltage sweeping ranges of −30/30 V, −20/20 V, and −10/10 V. b) Memory window width Δ*V* as a function of the maximum bottom gate voltage. c) Output curves at different *V*_BG‐P_ modulations. d) *I*_DS_ as a function of time for different states. e,f) Schematic representation of storage mechanism of the device with floating gate under opposite polarity gate voltage pulse modulations.

**Figure** **2** illustrates the electrical storage properties and functionality of the FG‐CSPT. Transfer curves of the device are measured by sweeping the bottom gate voltage (*V*_BG_) at a constant drain‐source voltage (*V*_DS_) of 0.01V. By modulating *V*_BG_ with different amplitudes, the device exhibits hysteresis characteristics, as shown in Figure 2a. The hysteresis transfer curves are measured within sweeping ranges of ±10, ±20, and ±30 V, respectively (sweeping direction of −10 V → 10 V → −10 V). The size of the hysteresis loop, a key parameter for evaluating the charge storage capability of the WSe_2_ FG‐CSPT memory, is extracted from the transfer curve and shown in Figure 2b. As the *V*_BG_ sweeping range increases, the amount of charge stored in the MLG layer increases, leading to a significantly expansion of the memory window width. And the memory window exhibits a linear correlation with the maximum *V*_BG_ magnitude, reaching a maximum value of 37.8 V. Figure 2c shows the output characteristic curves of the device in different storage states, measured after applying 1 ms bottom gate voltage pulse of −30 or 30 V. It can be observed that at *V*_DS_ = ±1 V, after a pulse modulation, the device current reaches 10^−5^ A, a significant improvement over the initial state (10^−11^ A), achieving a switching ratio of up to 10^6^. Simultaneously, the symmetry of *I*_DS_‐*V*_DS_ curves indicates excellent drain‐source symmetry in the WSe_2_ FG‐CSPT. Additionally, we investigated the transfer and output characteristics of the device under bottom gate voltage pulse modulations with varying amplitudes as shown in Figure S2 (Supporting Information). The non‐volatile electrical storage capability is demonstrated by the long‐time stability of the device's current in different states, as seen in Figure 2d (*V*_DS_ = 0.01 V). The output current amplitude remains nearly constant for up to 2000 s. Moreover, as shown in Figure 2e, we investigated and analyzed the floating gate storage mechanism of the FG‐CSPT. When a 30 V bottom gate voltage pulse is applied, electrons in WSe_2_ tunnel through the hBN layer into the floating gate MLG. WSe_2_ exhibits p‐type semiconductor behavior through the electrostatic doping effect of the stored electrons. Similarly, when a pulsed *V*_BG‐P_ of −30 V is applied, holes tunnel from the WSe_2_ layer into the MLG, inducing n‐type behavior. By modulating the bottom gate voltage pulses, we can switch WSe_2_ semiconductor behavior between modulation states dominated by different carriers, thereby demonstrating its bipolar properties.

**Figure 3:**
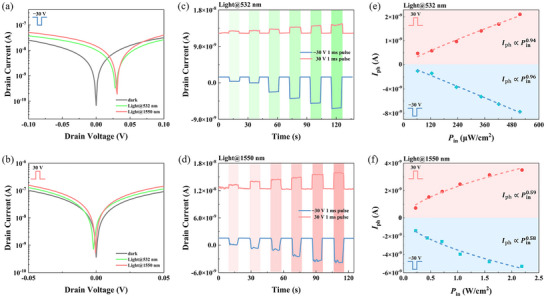
Optoelectronic characteristics of the floating‐gate phototransistor. a,b) *I*_DS_‐*V*_DS_ curves of the device under different modulation states and illumination. c,d) Photoresponse of the device to varying incident optical power for light of different wavelengths. The incident optical power density (*P*_in_) corresponding to 532 nm light are, in order, 62.5, 125, 234.4, 343.8, 421.9, and 515.6 µW cm^−2^, and *P*_in_ for 1550 nm is 0.22, 0.47, 0.72, 1.04, 1.59, and 2.19 W cm^−2^. e,d) Relationships between the net photocurrent *I*_ph_ and *P*_in_.

**Figure** **3** illustrates the photodetection characteristics of the device under 532 and 1550 nm illumination. To ensure stable photoresponse, the laser is focused at the drain terminal MLG/WSe_2_ Schottky junction via optical fiber coupling. Figure 3a,b display the output characteristic curves of the device under dark and light conditions. Notably, after applying *V*_BG‐P_ of ±30 V, the phototransistor exhibits photovoltaic behavior under both 532 and 1550 nm illumination. We measured the optoelectronic characteristics of the photodetector under different incident optical power densities (*P*_in_), and the results of 532 and 1550 nm light are shown in Figure 3c,d, respectively. As the incident optical power density increases, the device's responses to both wavelengths improve significantly. Additionally, we plotted the power‐dependent photocurrent and fitted these curves using the formula (Figure 3e,f). The values of α for 532 nm are close to 1, indicating linearity and a photoconduction mechanism. While exponents of α ≪ 1 are fitted for 1550 nm, as a result of the photogating effect in Ge.^[^ ^15^ ^]^ Furthermore, we calculated the responsivity (*R*) of the phototransistor under different illumination conditions and pulse modulations. Maximum responsivities of the device under −30 and 30 V pulse modulations (*V*_DS_ = 0.01 V) are −2.98 and 1.30 A W^−1^ under 532 nm illumination, and −1.08 and 0.57 mA W^−1^ for 1550 nm, respectively. The performance comparison between WSe_2_ FG‐CSPT and other floating‐gate phototransistors and retinal bionic devices is shown in Table S1 (Supporting Information). Compared with other devices, our floating‐gate phototransistor exhibits excellent performance due to its combination with the gate material germanium. This bipolar response behavior to both visible and infrared spectra enable the emulation of bipolar cells, shunting light signals into ON and OFF electric signals.

**Figure 4:**
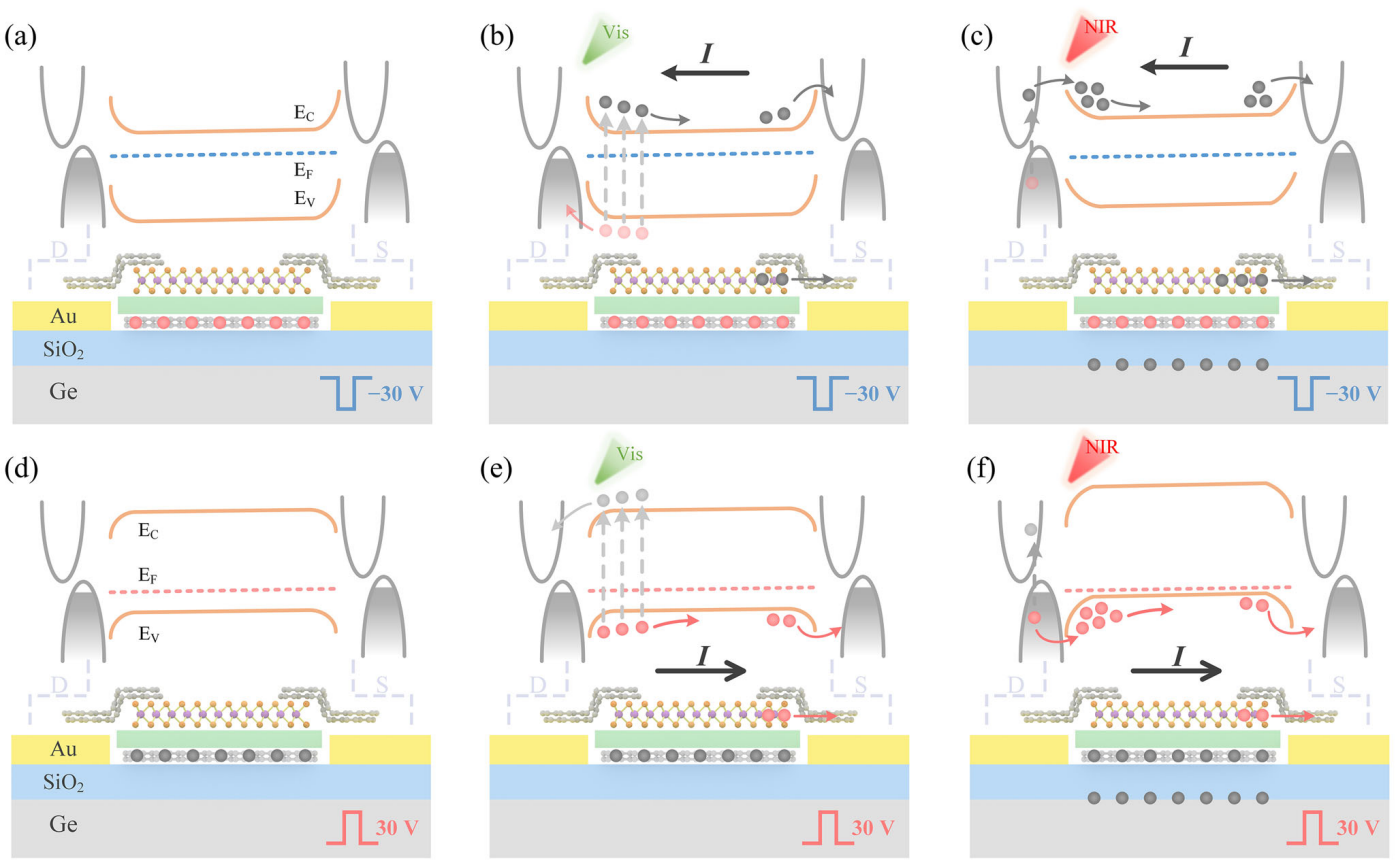
Mechanism explanation of FG‐CSPT. Energy band schematic of the device under a) dark condition, b) 532 nm illumination, c) 1550 nm illumination with V_BG‐P_ = −30 V and V_DS_ = 0.01 V. Energy band schematic of the device under d) dark condition, e) 532 nm illumination, f) 1550 nm illumination with V_BG‐P_ = 30 V and V_DS_ = 0.01 V.

**Figure** **4** details the mechanism of operation of the WSe_2_ floating‐gate phototransistor for a broad spectrum response. The device can achieve bipolar photoresponse across the Vis‐NIR spectrum under the modulations of bottom gate voltage pulses. As illustrated in Figure 4a–c, FG‐CSPT is modulated by a bottom gate voltage pulse of −30 V, while, in Figure 4d–f, a 30 V bottom gate voltage pulse (*V*_BG‐P_ = 30 V) is applied to the device. Notably, the above situations are discussed when the *V*_DS_ = 0.01V. Holes tunnel from the WSe_2_ layer to the floating gate MLG when *V*_BG‐P_ = −30 V, modulating the WSe_2_ material to n‐type. The energy band of WSe_2_ at the drain bends upward, where a significant electronic barrier is established between WSe_2_ and MLG (Figure 4a). Conversely, when *V*_BG‐P_ = 30 V, WSe_2_ is modulated to p‐type by the electrons stored in the floating gate. A smaller Fermi energy level difference between these two materials leads to a lesser extent band bending around the WSe_2_ and MLG interface as shown in Figure 4d. When a 532 nm laser is irradiated to the drain of the device (Figure 4b,e), the light absorption by WSe_2_ material produces a large number of photogenerated electron–hole pairs at the WSe_2_/MLG Schottky junction. Compared to the effect of the Schottky built‐in electric field, the drift effect of the photogenerated electron–hole pairs under the action of *V*_DS_ is much lower, since the applied drain‐source bias voltage is quite small (*V*_DS_ = 0.01 V). Therefore, it can be seen that photogenerated electrons are transported to the source due to the barrier when *V*_BG‐P_ = −30 V, generating a photocurrent with a direction pointing from source to drain (a negative photoresponse). Similarly, a positive response is generated under a bottom gate voltage pulse of 30 V, where holes are transported to the source. As shown in Figure S3 (Supporting Information), the influence of light illumination position on the bipolar photoresponse is investigated, demonstrating the critical role of the Schottky barrier in the response of the device.

The WSe_2_ FG‐CSPT response mechanism for 1550 nm is shown in Figure 4c,f. When 1550 nm light is irradiated on the device, due to the combination of photogating effect and Schottky barrier, the device exhibits bipolar photoresponse under bottom gate voltage pulse modulations. Germanium, due to its narrow bandgap, absorbs 1550 nm light and generates electron–hole pairs, playing an important role in the bipolar response of the device (shown in Figure S4, Supporting Information).^[^ ^16^ ^]^ Then, the photogenerated electrons are trapped by electron traps at the Ge/SiO_2_ interface. The presence of electron traps at the Ge/SiO_2_ interface is demonstrated in Figure S5 (Supporting Information). In addition, the initial band bending at the interface between MLG/SiO_2_ and SiO_2_/Ge under thermal equilibrium conditions caused by the difference in work functions between MLG and Ge materials can also lead to the accumulation of photogenerated electrons in Germanium at the SiO_2_/Ge interface.^[^ ^17^ ^]^ These trapped or accumulated electrons act as an external constant gate voltage, modulating the channel, which is known as the photogating effect. Meanwhile, the drain terminal MLG absorbs 1550 nm light for its inherent zero bandgap characteristic, and the photogenerated electrons/holes can cross the potential barrier between MLG and WSe_2_ due to the photo‐thermionic effect.^[^ ^18^ ^]^ Then the photogenerated carriers drift to the source under the influence of the built‐in electric field in the Schottky junction, which also leads to a bipolar response. In summary, under the combined effects of the photogating effect, photo‐thermionic effect, and Schottky barrier, the device exhibits a negative response at under a pulse of −30 V (Figure 4c) and a positive response when *V*_BG‐P_ = 30 V (Figure 4f), which is consistent with the result of 532 nm illumination. The bipolar response characteristics mentioned above provide new ideas and insights for practical and effective application of the WSe_2_ FG‐CSPT.

**Figure 5:**
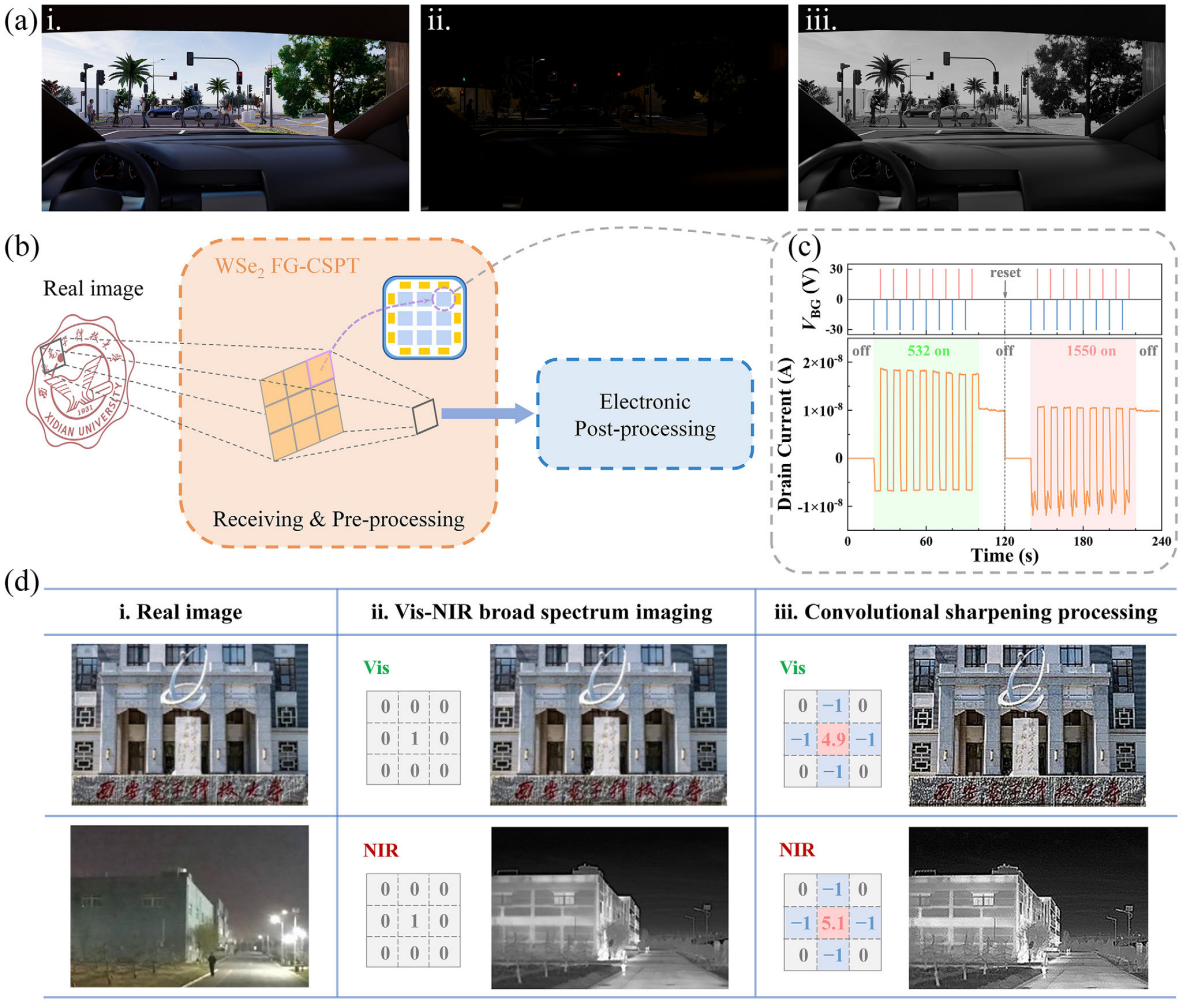
a) Schematic diagrams of the photodetector applications in vehicle imaging system. In normal daytime environment, schematic of (i) imaging effect of visible detector. In nighttime environment, schematic of (ii) vehicle scene, (iii) imaging effect of NIR detector. b) Schematics of advanced CNN system for image recognition processing based on the WSe_2_ FG‐CSPT. c) Simulation of neural biological functions of ON and OFF bipolar cells. d) Implementation of the FG‐CSPT for convolutional operation process in image perception processing. NIR images are supplied by Raytron Technology Co., Ltd. (Yantai, China).

Visible‐infrared retinal bionics, featuring dual‐band light detection and image process capabilities, hold significant potential for vehicle‐mounted imaging system applications. Visible photodetectors (usually silicon‐based photodetectors) deliver imaging in normal daytime conditions (**Figure** **5**a‐i), but lose their detection function in low‐visibility environments such as nighttime and foggy weather (Figure 5a‐ii). Due to their strong penetration and high imaging accuracy, infrared detectors have unique advantages in visual bionic chips, especially in low light and complex natural environments (Figure 5a‐iii).^[^ ^19^ ^]^ Ge‐based Vis‐NIR broad‐spectrum photodetector enables simultaneous detection of visible and infrared light, optimizing the detection range of traditional silicon sensors and providing new ideas for the development of high‐performance and multifunctional visual bionic chips. More importantly, by emulating the functionality of bipolar cells across both visible and NIR spectra, the FG‐CSPT photodetectors have the potential to be applied in convolutional neural network (CNN) image processing as image sensors and processors, as shown in Figure 5b. In the general CNN image recognition processing, after receiving the image information input by the optical frontend, the system needs to perform a convolution operation such as noise reduction/edge enhancement/sharpening through the processor. And then perform subsequent processing to finally recognition. However, floating‐gate phototransistors with bipolar photoresponse, can replace ordinary sensors and processors for image information perception and further convolution preprocessing, directly obtaining sharpened results. Compared to traditional CNN image processing systems, the introduction of our device can effectively save overall computing time and reduce system energy consumption.

Specifically, WSe_2_ FG‐CSPT could emulate the neural biology of ON and OFF bipolar cells by simply switch the polarity of gate voltage pulses under visible‐infrared broad‐spectrum. Figure 5c shows a negative photoresponse under a negative gate pulse, mimicking OFF‐bipolar cells, and a positive photoresponse under a positive gate pulse, resembling ON‐bipolar cells. *I*_DS_ shifted from −6.8 to 18.5 nA as *V*_BG‐P_ changed from −30 to 30 V under 532 nm illumination. After turning off the light and resetting, the current returned to its initial state. Similarly, for 1550 nm light, *I*_DS_ shifted from −10.6 to 10.7 nA. The response time and linear dynamic range (LDR) of the phototransistor emulating bipolar cells are shown in Figures S6–S8 (Supporting Information). The response time of the device for visible light is a few milliseconds, while a few microseconds for near‐infrared light. The maximum LDR of the device under 532 nm illumination is 24 dB. Based on the device's dual‐band bipolar weight parameters, we designed a 3 × 3 convolution kernel to enhance the effectiveness of image‐processing tasks. The real image is filtered into separate wavelength bands and divided into 3 × 3 sub‐images. In image processing, convolution is the element‐level multiplication and summation of convolution kernels and image pixels, mainly used for tasks such as image sharpening, edge detection, and feature extraction.

Here, the convolution operation is performed by sequentially processing each image pixel using the same device. The bipolar photocurrent generated from different light and bottom gate voltage pulse modulations is used to configure the convolution kernel for image sharpening processing. The specific test results and extraction method are shown in Figure S9 (Supporting Information). Figure 5d‐i shows conventional visible images during daytime and nighttime, respectively. Replacing the traditional Si photodetector with the Ge‐based photodetector results in a clearer nighttime image, as seen in Figure 5d‐ii. Furthermore, Figure 5d‐iii illustrates that by implementing a convolutional operation using the bipolar weight parameters of the Ge‐based FG‐CSPT, a significant sharpening effect is achieved, enhancing image clarity.

## 3 Conclusion

In summary, we present a floating‐gate phototransistor with a vertical WSe_2_/hBN/MLG/SiO_2_/Ge stacking structure, exhibiting non‐volatile gate‐controlled electrical and optoelectronic properties. The unique Schottky built‐in electric field at the drain/source and the photogating effect in Ge enable visible‐infrared broad‐spectrum bipolar photoresponse, emulating and expending neural biological functions of bipolar cells. Additionally, dual‐band light detection and image sharpening processing capabilities based on a convolution operation are achieved with the device's bipolar weights, significantly improving image quality. This study provides valuable insights for designing future visual bionic chips with low power consumption and perceptual computing capabilities.

## 4 Experimental Section

### Phototransistor Fabrication

A WSe_2_ floating‐gate phototransistor was prepared on a p‐type Ge substrate. First, a 300 nm thick SiO_2_ layer was deposited using plasma enhanced chemical vapor deposition. Second, the window in contact with the gate was etched using photolithography and reactive ion etching. Finally, Ni/Au (5 nm/50 nm) was deposited as a metal electrode using photolithography and electron beam evaporation. MLG, hBN, and WSe_2_ were stripped using mechanical stripping tape and sequentially transferred to the patterned substrate using polydimethylsiloxane employing a dry method.

### Characterization and Measurements

The electric and optoelectronic characteristics of the device were measured by a Keithley 4200A‐SCS Semiconductor Parameter Analyser in the dark and under 532 nm/1550 nm illumination. During measurement, the output light from the laser was coupled into an optical fiber, which was then illuminated onto the device. The output power of the 532 and 1550 nm laser from the fiber was calibrated using an optic power meter (PM100D, Thorlabs) connected to a silicon photodiode (S120C, Thorlabs) and an extended InGaAs photodiode (S148C, Thorlabs), respectively. The thicknesses of 2D materials are determined by an atomic force microscope (Park NX10). The Raman spectra were characterized using a confocal Raman system (WITec α300R) equipped with a 532 nm excitation laser.

## Conflict of Interest

The authors declare no conflict of interest.

## Supporting information



Supporting Information

## Data Availability

The data that support the findings of this study are available from the corresponding author upon reasonable request.
