# Supplementary material for: Ge‐Based Visible‐Infrared Bipolar Floating‐Gate Phototransistor for Broad‐Spectrum Retinal Bionics
Source: Adv Sci (Weinh). 2025 Sep 19;12(45):e12649. doi: 10.1002/advs.202512649 (PMC12677644; doi:10.1002/advs.202512649)
Supplement: Supplementary file 1 — Supporting Information [file ADVS-12-e12649-s001.docx]

Supporting Information

**Ge-Based Visible-Infrared Bipolar Floating-Gate Phototransistor for Broad-Spectrum Retinal Bionics**

*Qiancui Zhang, Yichi Zhang*, Xie Liu, Ningning Zhang, Tian Miao, Huiyong Hu, Liming Wang*, Zhangming Zhu*

**Device characterization.**





**Figure S1.** Atomic force microscopy (AFM) of WSe_2_ FG-CSPT. As shown in Figure S1c, the thicknesses of WSe_2_, hBN and floating gate MLG are about 13.8, 22.6 and 13.6 nm, respectively.

**Electrical device performance.**





**Figure S2.** (a-b) Transfer characteristics and (c-d) output characteristics of the device under various amplitude bottom gate voltage pulse modulations. It can be seen that carriers cannot effectively tunnel through hBN to the floating gate MLG when *V*_BG-P_ = ±10 V. While modulation with an amplitude of 20/30 V, carriers in channel can tunnel to the floating gate and be stored. Compared with the initial state, transfer curves show significant left/right shift, and output curves acquire higher *I*_DS_.

**Optoelectronic characteristics.**

In order to demonstrate the effect of asymmetric illumination conditions on the bipolar photoresponse of the floating-gate device, the curve of the device’s photocurrent over time under three positions of illumination were studied. It was found that when the position of the light is changed to global or source illumination, the bipolar response of the device decreases or even reverses. It is indicated that the device’s photoresponse is highly sensitive to the illumination position, further proving the existence of a dominant role of Schottky barrier at the WSe_2_/MLG junction in the transport direction of photogenerated carriers.





**Figure S3.** Sensitivity of WSe_2_ FG-CSPT to 532 nm illumination position. The photoresponse of the device (a) under partial illumination at the drain, (b) under global illumination, (c) under partial illumination at the source, respectively.





**Figure S4.** The bipolar photoresponse of (a) germanium-substrate and (b) silicon-substrate of the floating-gate phototransistors. The near-infrared detection performance of Ge-based device is significantly better than that of Si-based device, indicating that germanium plays an enhancing role in the NIR bipolar response of the device.





**Figure S5.** Study of transfer characteristics of the floating-gate composite structure phototransistors with diverse substrates. Transfer characteristics of (a-b) Ge substrate and (c-d) Si substrate (*V*_DS_ = 0.1 V). A higher drain-source bias voltage can effectively reduce the contact barrier between MLG and WSe_2_ at two terminals of the device, weakening the influence of barrier and thus highlighting the role of the photogating effect on photoresponse.

**Emulating the bionic characteristics of bipolar cells.**

The response time and linear dynamic range (LDR) of the device are shown in Figures S6-8. Under 532 nm illumination, the rise times (*t*_r_) of the device are 10.8 ms (*V*_BG-P_ = −30 V) and 6.3 ms (*V*_BG-P_ = 30 V), and the fall times (*t*_f_) are 9.1 and 5.7 ms, respectively. For 1550 nm light, *t*_r_ = 15.7 μs and *t*_f_ = 9.4 μs when *V*_BG-P_ = −30 V, while *t*_r_ and *t*_f_ both are 15.7 μs when *V*_BG-P_ = 30 V. In the visual system, the way to process external information is to convert sensory stimuli into digital spikes, and neurons can emit spikes with a precision of a few milliseconds relative to the stimulus time.^[1]^ The floating-gate phototransistor simulates the process of human retina sensing and transmitting signals. It can be seen that the response time of the device to visible light is comparable to that of neurons, and the response time of near-infrared light is significantly better than that of actual biological systems.





**Figure S6.** The response time of the phototransistor for 532 nm. The solid line represents the fitting to the data.





**Figure S7.** The response time of the device under 1550 nm illumination.

The linear dynamic range (LDR) is defined as the linear proportional relationship between the output photocurrent and the input optical signal within the range of optical power margin:

LDR = 20 × log$\frac{P_{max}}{P_{min}}$

where *P*_max_ and *P*_min_ are the upper and lower limits of the optical power in a particular range.^[2]^ LDR of the device are 20.3 dB (*V*_BG-P_ = −30 V) and 24.3 dB (*V*_BG-P_ = 30 V), respectively. It is not significantly different from other reported photodetectors with linear dynamic ranges of several tens of decibels.^[3]^ However, there is still a significant gap compared to the dynamic range of about 200 dB for the human eye.^[4]^





**Figure S8.** Linear dynamic range of the phototransistor under 532 nm illumination with various incident optical power density. The dashed line represents linear fitting to the data.

**Extraction of convolutional kernel in image sharpening.**

Inspired by the bipolar photoresponse of WSe_2_ FG-CSPT to visible-infrared broad-spectrum, the bipolar response characteristics of the device can be applied to image sharpening based on convolution operation. The bipolar photocurrent is related to the bottom gate voltage pulse. By adjusting the bottom gate voltage pulse, the convolution kernel required for image sharpening can be configured to achieve image processing.

In the visible/near-infrared band, the value of convolution kernel is obtained by comparing the amplitude of the bipolar photocurrent of the device. Specifically, the ratio of the negative photoresponse current to the positive photoresponse current of the device determines the ratio of the weight values at the center and around of the convolution kernel. The amplitude ratio of bipolar photoresponse of the device is 4.9 under 532 nm illumination (corresponding kernel is [0, −1, 0], [−1, 4.9, −1], [0, −1, 0]), and 5.1 for 1550 nm (kernel is [0, −1, 0], [−1, 5.1, −1], [0, −1, 0]).





**Figure S9.** Bipolar photocurrent generated by the device under different bottom gate voltage pulse modulations in visible and near-infrared light bands. The amplitude ratio of bipolar photoresponse of the device is 4.9 for 532 nm illumination, and 5.1 for 1550 nm.

**Comparison with other devices.**

**Table 1.** Comparison of the performance of WSe_2_ FG-CSPT with other floating-gate phototransistors and retinal bionic devices.

| Device structure | Switching  ratio | Retention performance (s) | Wavelength (nm)* | Responsivity | Reference |
| --- | --- | --- | --- | --- | --- |
| WSe_2_/hBN/MLG/SiO_2_/Si  WSe_2_/hBN/Gr/ SiO_2_/Si  MoS_2_/hBN/CsPbBr_3_ QDs  /hBN/Gr  MoS_2_/hBN/Gr/SiO_2_/Ge  ReSe_2_/hBN/Gr/SiO_2_/Si  WSe_2_/hBN/MLG/SiO_2_/Ge | 10^4^  10^2^  10^7^  10^6^  10^4^  10^6^ | 1 × 10^3^  1.2 × 10^3^  3 × 10^3^  1 × 10^3^  1 × 10^3^  2 × 10^3^ | 532 (bipolar)  637 (bipolar)  405 (positive)  532 (positive)  1550 (negative)  532 (negative)  532 (bipolar)  1550 (bipolar) | 0.45 A/W (*V*_DS_ = 0)  2.76 A/W (*V*_DS_ = 0)  Not mentioned  12.7A/W, −24 mA/W  (*V*_DS_ = 0.05 V)  Not mentioned  1.30A/W, −1.08 mA/W  (*V*_DS_ = 0.01 V) | [5]  [6]  [7]  [8]  [9]  This work |

*The words bipolar/positive/negative in parentheses after the wavelength indicate that the device exhibits bipolar/positive/negative photoresponse at the corresponding wavelength.

**References**

[1] T. Baden, F. Esposti, A. Nikolaev, L. Lagnado, *Current Biology* **2011**, *21* (22), 1859.

[2] C. Li, H. Wang, F. Wang, T. Li, M. Xu, H. Wang, Z. Wang, X. Zhan, W. Hu, L. Shen, *Light: Science & Applications* **2020**, *9* (1), 31.

[3] a) M. Dai, H. Chen, F. Wang, M. Long, H. Shang, Y. Hu, W. Li, C. Ge, J. Zhang, T. Zhai, *ACS nano* **2020**, *14* (7), 9098; b) L. Wei, Z. Wu, Y. Wei, C. Li, Z. Fu, J. Han, X. Yang, J. Xie, Z. Tian, H. Zhou, *Advanced Functional Materials* **2024**, *34* (52), 2411736; c) S. Zhou, S. Wen, H. Fan, Y. Wei, Y. Yin, C. Lan, C. Li, Y. Liu, *ACS Photonics* **2024**, *11* (4), 1810; d) C. Hong, Y. Tao, V. K. Dat, J.-H. Kim, *npj Flexible Electronics* **2025**, *9* (1), 13; e) L. Jian, S. Zhang, W. Gao, Y. Sang, Y. Sun, N. Huo, Z. Zheng, M. Yang, *Applied Physics Letters* **2024**, *124* (9); f) Y. Lu, T. Chen, N. Mkhize, R.-J. Chang, Y. Sheng, P. Holdway, H. Bhaskaran, J. H. Warner, *ACS nano* **2021**, *15* (12), 19570.

[4] B. Hoefflinger, *SPRINGER SERIES IN ADVANCED MICROELECTRONICS* **2007**, *26*, 1.

[5] Z. Han, Y. Zhang, Q. Mi, J. You, N. Zhang, Z. Zhong, Z. Jiang, H. Guo, H. Hu, L. Wang, *ACS nano* **2024**, *18* (43), 29968.

[6] Z. Wang, J. Jian, Z. Weng, Q. Wu, J. Li, X. Zhou, W. Kong, X. Xu, L. Lin, X. Gu, *Advanced Science* **2025**, 2417300.

[7] W. Ouyang, Q. Zhang, J. Chen, X. Luo, X. Wang, Y. Chen, F. Yang, Q. Nie, Q. Liu, F. Liu, *Advanced Science* **2025**, 2502694.

[8] B. Wang, N. Zhang, J. You, X. Wu, Y. Zhang, T. Miao, Y. Liu, Z. Jiang, Z. Zhong, H. Sun, *InfoMat* **2025**, e12661.

[9] W. Li, T. Mu, P. Li, S. Zhang, P. Sun, J. He, X. Hu, Y. Wang, X. Gan, S. Wang, *Advanced Functional Materials* **2025**, 2425359.
